# Sickness Response Symptoms among Healthy Volunteers after Controlled Exposures to Diesel Exhaust and Psychological Stress

**DOI:** 10.1289/ehp.1002631

**Published:** 2011-02-17

**Authors:** Robert J. Laumbach, Howard M. Kipen, Kathie Kelly-McNeil, Junfeng Zhang, Lin Zhang, Paul J. Lioy, Pamela Ohman-Strickland, Jing Gong, Alexander Kusnecov, Nancy Fiedler

**Affiliations:** 1 University of Medicine and Dentistry of New Jersey–Robert Wood Johnson Medical School, Piscataway, New Jersey, USA; 2 University of Medicine and Dentistry of New Jersey–School of Public Health, Piscataway, New Jersey, USA; 3 Rutgers, the State University of New Jersey, Piscataway, New Jersey, USA

**Keywords:** diesel exhaust, Gulf War illness, psychological stress, sickness response, symptoms

## Abstract

Background: Interactions between acute exposures to environmental chemical contaminants and psychological stress may be important in situations where they are likely to co-occur, ranging in intensity from daily urban living to participation in war. Modification of symptomatic responses by stress may play a role in medically unexplained symptoms attributed to low-level chemical exposures.

Objectives: We hypothesized that the combination of exposure to diesel exhaust (DE) and acute psychological stress would cause sickness responses in healthy volunteers. Moreover, these responses would be greater in individuals with self-reported prior chemical odor intolerance.

Methods: One hundred adult subjects underwent 1-hr exposures to diluted DE and clean air control. Half of the subjects performed a public-speaking stressor task during the exposures. Subjects completed questionnaires to determine their Chemical Odor Intolerance Index score. Plasma cortisol, end-tidal carbon dioxide, and the severity of 35 symptoms were measured at time points before and after the exposures.

Results: Subjects exposed to DE demonstrated small but statistically significant increases in severity for several symptom categories, including sickness response and upper respiratory, central nervous system, and total symptoms. The psychological stressor did not increase symptom severity independently or via interaction with DE. Subjects with prior self-reported chemical intolerance had more severe sickness response symptoms from DE.

Conclusions: These results suggest that exposure to DE can cause acute sickness response symptoms and that these symptoms are also associated with increased levels of self-reported chemical intolerance. The results did not confirm our hypothesis that an acute stressor would increase sickness response symptom severity during the exposure.

Interactions between exposures to psychological stress and environmental contaminants may be important in many settings in which exposures to stress and pollution are likely to co-occur, ranging in intensity from daily urban living to participation in wartime activities. Recent reports of effect modification by exposure to psychosocial stress include effects on associations between air pollution and asthma incidence and exacerbation among children (Chen et al. 2008; Clougherty et al. 2007) and between exposure to lead and hypertension among older adults (Peters et al. 2007). Outcomes of potential interest include not only physiological effects but also symptomatic responses. Psychological stress has been invoked as a potential modifier of the symptomatic responses that are characteristic of medically unexplained symptoms (MUSs) attributed to low-level chemical exposures, including Gulf War illness, multiple chemical sensitivity, and sick building syndrome (Kipen and Fiedler 1999, 2002a, 2002b).

The lack of specificity among MUSs has given rise to a wide range of hypothesized etiologies, including exposure to various toxicants, psychological stress, and behavioral and psychological conditions (Gronseth and Gronseth 2005; Kipen and Fiedler 2002b). Many MUSs attributed to environmental exposures are consistent with those of the “sickness response,” a behavioral correlate of the acute-phase response to actual or threatened injury (Dantzer 2001). Sickness response symptoms include fatigue, lowered pain threshold, social withdrawal, loss of appetite, depressed mood, cognitive disturbances, and other illness symptoms (Dantzer et al. 1993; Maier and Watkins 1998). Exposures to air pollutants and acute psychological stressors can independently cause elements of the acute-phase response in animals and humans, suggesting that coexposure may have additive or synergistic effects on sickness response symptoms (Maier and Watkins 1998; Salvi et al. 1999; Watkins and Maier 1999).

Effects of coexposure to air pollutants and stress, and their interactions, are likely to depend on the timing, duration, and intensity of exposures (Clougherty and Kubzansky 2009). Ferguson and Cassady (1999) proposed a “bioassociative” model of Gulf War illness in which toxic exposures during the war, such as diesel exhaust (DE), in combination with physical and acute psychological stress, caused an acute sickness response (Ferguson and Cassaday 1999, 2001). This unconditioned sickness response was associated with odors that are later experienced in the home environment, triggering persistent, conditioned sickness responses among susceptible individuals (Ferguson and Cassaday 2001). This model might also explain other MUSs attributed to environmental exposures, in which an initial higher-level exposure is often reported (Miller 1999). In the present study, we focused on whether combined exposure to acute stress and DE could cause an acute, symptomatic sickness response (the unconditioned response in Ferguson and Cassaday’s model).

Observational and controlled-exposure studies have demonstrated associations between DE and eye, nose, and respiratory irritation, as well as symptoms consistent with the sickness response, including nausea, fatigue, impaired memory, lack of concentration, vertigo, and abdominal discomfort (Gamble et al. 1987; Kilburn 2000; Rudell et al. 1996, 1999; Scheepers and Bos 1992). DE is a mixture of hundreds of compounds, many of which are known toxicants, including potent irritants such as formaldehyde and other carbonyls. Induction of a sickness response by tissue injury and/or inflammation is one possible explanation for generalized illness symptoms from inhalation of DE (Maier and Watkins 1998; Watkins and Maier 1999).

Any interactions between DE and stress are likely to be influenced by individual susceptibility. Illness symptoms from common chemical exposures, measured by self-report as the Chemical Odor Intolerance Index (CII), vary greatly among individuals (Szarek et al. 1997). In a cross-sectional study, higher CII scores were associated with poorer global health among Gulf War veterans (GWVs) (Bell et al. 1998). However, associations between CII scores and symptoms from measured levels of a common air contaminant have not been previously validated in a controlled-exposure setting.

We hypothesized that a short-term, controlled exposure to DE would cause increased symptoms consistent with the sickness response and that these effects would be augmented by simultaneous exposure to an acute psychological stressor. Furthermore, we hypothesized that individuals with higher CII scores would have greater responses to combined stress and DE exposures. To test these hypotheses, we conducted a controlled-exposure study of diluted DE and a stressor (a standardized public-speaking task) among healthy volunteers.

## Materials and Methods

*Subjects.* Subjects were recruited from the University of Medicine and Dentistry of New Jersey (UMDNJ)–Robert Wood Johnson Medical School and Rutgers University community and surrounding New Jersey suburbs through postings and advertisements in newspapers. One-hundred sixteen healthy, nonsmoking subjects met screening criteria for the study and completed a physical examination. Nineteen of the 116 (16%) subjects did not complete the study [3 did not complete the CII questionnaire, 4 dropped out after physical exam, and 12 did not complete the second exposure session, among whom only one subject had an adverse reaction to the first exposure (a vasovagal response)]. Ninety-seven subjects (35 female, 62 male) with a mean ± SE age of 24 ± 0.58 years and education of 15.9 ± 9.28 years completed the study. Subjects were primarily Asian or Caucasian (Asian, 38%; Caucasian, 36%; African American, 7%; Hispanic, 17%; other, 2%). Subjects who reported any of the following health conditions were excluded: neurological disease or brain injury, migraine headaches, stroke or cardiovascular disease, cancer, pulmonary disease including asthma, liver or kidney disease, endocrine disease, hypertension, multiple chemical sensitivity with significant illness behavior or disability, and major psychiatric conditions, including psychoses, bipolar disorder, alcoholism, or drug abuse. Current smokers and pregnant or lactating women were not included. History of allergic rhinitis and/or hay fever was noted for all subjects. All recruitment and testing procedures were reviewed and approved by the Institutional Review Board of UMDNJ.

*Chemical Odor Intolerance Index.* The CII is a scale with documented reliability and factorial validity that measures individual differences associated with feeling ill in response to chemical mixtures (Baldwin et al. 1997; Bell et al. 1995, 1996; Szarek et al. 1997). Subjects rated their history of self-reported illness associated with five everyday chemicals (perfume, pesticide, paint, car exhaust, and new carpet) on a 5-point Likert scale, which was summed with a range of possible scores from 5 to 25.

*DE exposure.* Each subject participated in one 60-min exposure to a filtered-air control condition (“clean air,” CA) and one 60-min exposure to diluted DE [300 μg/m^3^; particulate matter ≤ 2.5 μm in aerodynamic diameter (PM_2.5_)] in random order on separate mornings at least 1 week apart. The exposures were conducted in the Environmental and Occupational Health Science Institute’s Controlled Environment Facility (CEF), a 25-m^3^ stainless steel–lined chamber in which PM_2.5_ concentrations, temperature, and humidity were controlled. The exposure system and methods used to characterize the exposures have been previously described (Laumbach et al. 2009). Briefly, the DE was generated by a 5,500-W electricity generator (model YDG 5500EE; Yanmar, America Corp., Adairsville, GA) that contained a 406-cc–displacement air-cooled engine. The engine was maintained at 100% of rated capacity during the DE exposure session. A desired amount of DE was introduced to the CEF air delivery stream to achieve a targeted DE PM_2.5_ concentration of approximately 300 μg/m^3^ within the CEF. This concentration was monitored and maintained throughout a DE exposure session.

*Stressor.* Half of the subjects were randomized to perform the stressor, a public-speaking task, midway through the DE and CA exposures. The stressor was performed at 25 min after the start of the exposure to enable us to distinguish stress effects due to the onset of the exposure from stress effects due to the stressor. The subject delivered a 4-min speech after a 4-min silent preparation period. To enhance the stressfulness of the procedure, subjects were told that the research technician was evaluating the speech as it was given, that three staff members would evaluate a videotape of the speech later, and that an additional stipend of $10 would be awarded for good performance. All subjects received the added stipend regardless of performance. Two distinct speech scenarios were used as described in Al’Absi et al. (1997). Subjects who did not complete the stressor were instructed to complete simple arithmetic problems during the same time frame as for the public-speaking task. They were told to do their best and to work at their own pace with no time limit.

*Symptom questionnaires.* Each of 35 symptoms was rated on a labeled magnitude scale (Green et al. 1996), ranging from 0 (barely detectable/no sensation) to 5 (weak) to 15 (moderate) to 100 (strongest imaginable). Symptoms were chosen based on our previous work assessing the health effects of indoor air pollutants and diesel vapor (Fiedler et al. 2004), previous studies of DE (Rudell et al. 1996), and symptoms associated with the sickness response (Maier and Watkins 1998). These symptoms were grouped into *a priori* categories (subscales): *a*) sickness response comprised fatigue, drowsiness, difficulty concentrating, nausea, stomachache, body temperature, or body ache; *b*) eye irritation comprised eye irritation or runny/watery eyes; *c*) upper respiratory response comprised nose irritation, dryness, or itching, throat irritation, nasal congestion, sneeze, or choking; *d*) lower respiratory response comprised coughing, chest tightness, shortness of breath, wheeze, or pain on deep inspiration; *e*) anxiety response comprised responses such as “I feel jittery in my body,” “I feel nervous,” heart palpitations, chest pain, “I feel tense,” or “I am worried”; *f*) central nervous system (CNS) response comprised headache, dizziness, lightheadedness, or feeling disoriented/confused; *g*) somatic control response (symptoms not typically associated with air pollution exposures) (Fiedler et al. 2004) comprised skin irritation or dryness, ear ringing, sweating, numbness/tingling, leg cramps, or back pain.

*Procedure.* Prospective subjects who contacted the research office gave informed consent to complete a telephone questionnaire that screened for inclusion/exclusion criteria. After informed consent, subjects completed a medical history questionnaire, physical examination, routine blood chemistries, spirometry, electrocardiogram, and blood pressure to rule out any medical conditions that would preclude participation. Subjects were introduced to the CEF, where they were given an orientation to the questionnaires and procedures, and they practiced the nasal lavage and induced sputum procedures. This session was used to reduce practice and novelty effects in subsequent sessions.

Subjects were randomly assigned to the order of exposure conditions; half of the subjects were assigned exposure conditions with a stressor, and the remaining half of the subjects did not perform the stressor. The subjects and the research technicians were blinded to the exposure conditions.

Each experimental session was approximately 110 min in duration and occurred in the same time in the morning to control for circadian rhythm. On the day before each testing session and on the day of the testing session, subjects were asked not to use caffeine or alcohol. Subjects had to be free of any upper respiratory illness (either infection or allergy) and not using medication for allergies or other respiratory conditions for at lease 1 week before an exposure session.

**Figure 1:**
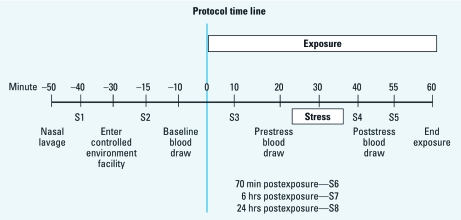
Time line for experimental procedures. Time 0 is start of DE or CA exposure. The symptom questionnaire was administered at time points labeled S1–S8.

One or two subjects were tested during an exposure session. All exposure sessions started at 0830 hours. Subjects completed the symptom questionnaire to establish symptoms 40 min before the start of exposure (baseline; S1 in Figure 1), 15 min before start of exposure (baseline; S2), and 10 min (S3), 40 min (S4), 55 min (S5), 70 min (S6), 6 hr, (S7), and 24 hr after the start of exposure. For additional details on study procedures, which included serial collections of venous blood for cortisol analysis, measurement of end-tidal carbon dioxide (CO_2_), and electrocardiograph recording, see Supplemental Material (doi:10.1289/ehp.1002631).

*Statistical analyses.* A total symptom severity score was created for each time point by summing ratings for all symptoms. Scores for subscales of symptoms were created by summing ratings for each symptom within the subscales. The effects of exposure on changes in symptom severity rating over time (hypothesis 1) were analyzed via mixed linear models (with Proc Mixed in SAS; version 9.2, SAS Institute Inc., Cary, NC), with the change in severity from baseline S2 to another time point (during or after exposure) as the response. Time was included as a categorical variable. In addition, a cross-product between exposure and time captured differences in changes in symptoms between exposures. Type 3 score tests of the cross-products were used to measure the effect of exposure. This analysis was first completed for the total symptoms and then for each classification of symptoms. An uncorrected α-level of 0.05 was used for statistical testing. Uncorrected α-values are reported with statistical significance after Bonferroni correction noted for each group of multiple comparisons. The uncorrected α-levels are reported for tests of the sickness response symptoms, the main hypotheses of the study.

To examine whether psychological stress modified the exposure effect (hypothesis 2), an interaction between exposure, stress, and time was added to the model described above. Finally, to examine whether chemical intolerance (measured as CII score) affected the exposure–stress effect (hypothesis 3), the model above was modified further to include a four-way interaction between exposure, stress, CII score, and time.

## Results

*Physical and chemical characterization of exposures.* Physical and chemical characterization of major components of the exposure atmospheres (Table 1) showed that mean PM_2.5_ mass concentration in the CEF during the 1-hr DE exposure condition was within 10% of our target of 300 μg/m^3^, with a range of 210–337 μg/m^3^. Mean carbon monoxide, nitric oxide, and nitrogen oxide concentrations were 3.7 ppm, 3.17 ppm, and 0.21 ppm, respectively. More extensive characterization of two samples of the DE showed the presence of formaldehyde at 196 ± 1.28 μg/m^3^, acetaldehyde at 79.6 ± 5.72 μg/m^3^, other carbonyls, and various polycyclic aromatic hydrocarbons that were typical of DE. Temperature and humidity were maintained at 72 ± 0.5°F and 40 ± 2% relative humidity, respectively.

**Table 1:** Summary of CEF exposure conditions during exposure sessions.

Table 1. Summary of CEF exposure conditions during exposure sessions.										
Exposure condition		PM_2.5_ mass (μg/m^3^)		PM number (no./cm^3^)		Carbon monoxide (ppm)		Nitric oxide (ppm)		Nitrogen dioxide (ppm)
DE exposure (*n* = 79)										
Mean		277		64,111		3.7		3.17		0.21
SD		20.3		16,718		0.85		1.04		0.28
Range		210–337		33,097–107,235		2.20–6.10		1.00–6.3		0.07–1.96
CA exposure (*n* = 78)										
Mean		6.69		4,095		0.93		0.01		0.007
SD		6.20		2,487		0.19		0.02		0.005
Range		1.0–31		745–16,538		0.50–1.93		0.00–0.13		0.00–0.03

We confirmed a physiological stress response by a mean 12% increase in plasma cortisol from prestress to poststress time points among the stressed group, compared with no change for the unstressed group (*p* = 0.02).

**Figure 2:**
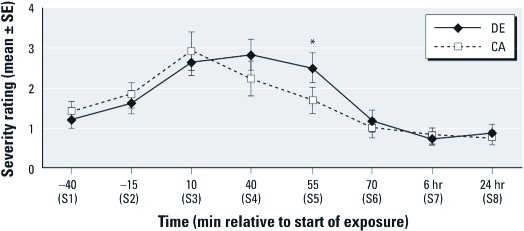
Mean sickness response symptom severity (± 1 SE) at each measurement time for DE and CA control exposure sessions: 0 (no sensation) to 100 (strongest imaginable). Mean severity is in the “weak” range (1–4); see “Discussion.” Exposure × time interaction for overall change in symptom severity from baseline (–15 min) to times 10, 40, and 55 min was statistically significant (*F* = 2.97; df = 4, 692; *p* = 0.019). *p < 0.01 for effect of DE on change in severity from –15 min to time 55 min after the start of exposure.

**Figure 3:**
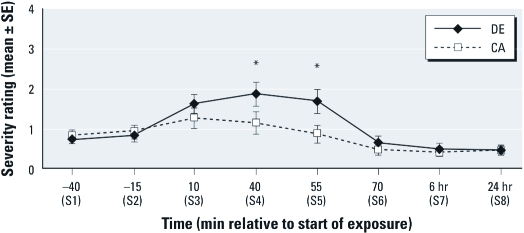
Mean total symptom severity (± 1 SE) at each symptom measurement time for DE and CA control exposure sessions: 0 (no sensation) to 100 (strongest imaginable). Mean severity is in the “weak” range (1–4); see “Discussion.” Exposure × time interaction for overall change in symptom severity from baseline (–15 min) to times 10, 40, and 55 min was statistically significant (*F* = 3.69; df = 4, 692; *p* = 0.0055). *p < 0.001 for effect of DE on changes in severity from times –15 min to 40 min and from –15 min to 55 min after the start of exposure.

*Hypothesis 1 (exposure × time): relative to CA, DE exposure will cause increased sickness response symptoms.* We partially confirmed hypothesis 1. After controlling for baseline S2 (–15 min), we observed a significant overall increase during exposure (at 10, 40, 55 min) in mean sickness response symptom scores for DE relative to CA exposure (*p* = 0.019). We observed a significant exposure × time interaction at 55 min (*p* < 0.01) (Figure 2), with greater sickness response symptoms during DE relative to CA exposure. Sickness response symptoms were increased at 10 min for both DE and CA exposure but remained elevated during DE exposure at 40 and 55 min, whereas sickness symptoms scores returned to baseline S2 levels by 55 min with exposure to CA. We observed a similar overall increase in total symptom severity with DE relative to CA during the exposure (*p* = 0.0055), with significantly increased symptoms at 40 min and 55 min (both *p* < 0.001) (Figure 3). In exploratory analyses, we saw overall increases in symptom severity during exposure for eye irritation (*p* = 0.035), lower respiratory (*p* = 0.010), upper respiratory (*p* = 0.0012), and CNS (*p* = 0.0005) symptom categories. With Bonferroni correction for these exploratory analyses, overall increases in total symptom severity, upper respiratory, and CNS symptoms remained significantly increased for DE compared with CA. Among the individual symptoms, eye irritation was significantly greater at 40 and 55 min, whereas lower and upper respiratory and CNS symptoms were significantly greater at 10, 40, and 55 min for the DE relative to CA exposure. Moreover, lower respiratory symptoms persisted 10 min after conclusion of the DE exposure (70 min).

*Hypothesis 2 (exposure × stress × time): psychological stress will increase symptoms in the DE relative to CA exposure condition.* Contrary to our hypothesis, exposure to the stressor did not augment the increases in the symptom severity scores that we observed with DE compared with CA. We found no significant interactions between exposure and stress for any of the symptom categories (data not shown).

**Figure 4:**
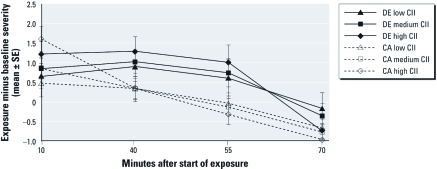
Differences in mean sickness response symptom severity (± 1 SE) between baseline S2 and 10, 40, 55, and 70 min after start of exposure for DE and CA among individuals grouped as low, medium, and high CII score (25th, 50th, and 75th percentiles, respectively). *y*-Axis values are the differences between S2 and time points 10, 40, 55 and 70 min. Exposure ended at 60 min. Mean sickness symptom severity ratings for each group and time point were derived from mixed linear models. CII score × exposure × time interaction was statistically significant (*F* = 2.76; df = 3, 664; *p* = 0.041).

*Hypothesis 3 (exposure × stress × CII score × time): subjects with higher CII scores will have greater symptom severity in response to combined exposure to stress and DE.* The mean ± SD CII score was 6.6 ± 1.9 (interquartile range, 5–8). We did not confirm the hypothesis for the interaction of exposure, stress, CII score, and time (data not shown). However, we may have had insufficient statistical power to detect important effects in this four-way interaction. Ignoring stress, we observed progressively elevated, S2 baseline–corrected, sickness response scores with DE exposure for 25th, 50th, and 75th percentile groups of CII scores at 40 and 55 min (Figure 4). With CII score as a continuous variable in the statistical model, the interaction between exposure, CII score, and time was significantly different for the sickness response symptoms category (*p* = 0.041). Higher CII score was associated with similar, but smaller, augmentation of DE effects on total symptom severity and somatic symptom severity at 40 and 55 min, but these effects were not statistically significant (data not shown). CII score did not interact with stress to alter the severity of any symptom category at any time point (data not shown).

## Discussion

Compared with a CA control exposure, a 1-hr exposure to DE caused increased sickness response symptoms among healthy young adults. The severity of these symptoms was greater in subjects who reported higher levels of chemical intolerance (higher CII scores) before exposures. However, the results did not confirm our hypothesis that an acute stressor would increase sickness response symptom severity during exposure to DE.

To our knowledge, this is the largest controlled-exposure study evaluating symptoms associated with exposure to DE and validating CII score as a predictor of illness behavior. “Chemically intolerant” individuals are known to report numerous symptoms that they associate with chemical exposure, but the lack of specificity suggests a generalized reactivity to external stimuli perceived by the individuals to be noxious or harmful. Therefore, the present results are noteworthy in differentiating a more systemic sickness response from the localized respiratory and eye irritation that is commonly associated with DE exposure. Although we did not define “ill” in the CII questionnaire, CII score predicted increased reporting of symptoms that we selected *a priori* as being reflective of sickness responses that manifest as “nonspecific” symptoms likely to be interpreted as “illness” (fatigue, drowsiness, difficulty concentrating, nausea, stomachache, body ache, and body temperature). The utility of CII as an instrument for predicting responses to noxious chemical exposures such as DE has not been previously demonstrated in a controlled-exposure study.

As hypothesized, DE exposure was associated with increased sickness response symptoms (Figure 2). We also found increases in upper and lower respiratory, eye irritation, central nervous system, and total symptom (Figure 3) severity during DE versus CA control exposures, although only upper respiratory, CNS, and total symptoms remained statistically significant after Bonferroni correction. A strong correlation between upper respiratory symptom severity and sickness response symptom severity (Pearson *r* = 0.43, *p* <0.0001, for changes from baseline S2 through the end of exposure) suggests that local tissue injury or inflammation caused by irritants might mediate the effects of DE on systemic sickness response symptoms, as has been suggested by others (Ferguson and Cassaday 1999; Maier and Watkins 1998; Watkins and Maier 1999).

Overall, the degree of irritation and other respiratory symptoms during DE exposure in our study was reported as mild, with mean severity levels near the “weak” descriptor on the symptom severity scale, although some subjects reported symptoms at the “moderate” level (data not shown). These symptomatic responses are consistent with those reported in earlier controlled exposures to DE. Rudell et al. (1996, 1999) also found modest increases in eye, nose, and throat irritation and headache during DE exposure at the same PM-standardized concentration and duration as we used here. The increase in respiratory and CNS symptoms is also consistent with reports of associations between exposure to DE and respiratory symptoms in occupational settings, although potentially confounding exposures were present in those studies (Gamble et al. 1987; Kilburn 2000). The observation that DE can cause symptoms of respiratory irritation is not surprising, considering that the complex DE mixture includes several strong respiratory irritants, including nitrogen dioxide, formaldehyde, and other aldehydes. Headache, included in our *a priori* grouping of CNS symptoms, has been reported in association with sensory irritant exposures (Otto et al. 1990) and may be associated with triggering of mucosal irritant receptors, but evaluation of this mechanism was beyond the scope of our study.

Contrary to one of our hypotheses, acute psychological stress during the DE exposure did not enhance sickness response symptoms. The public-speaking task, which has been shown to produce reliable physiological stress in experimental settings (Al’Absi et al. 1997), did induce a significant increase in plasma cortisol 20 min after the stressor task. Stress has been associated with increased symptom reporting in other settings in which symptoms have been attributed to chemical exposure, such as health complaints in buildings (Crawford and Bolas 1996; Hodgson 1995; Mendell 1993). However, the public-speaking stressor task used in the present study was also not associated with increased symptoms in our earlier controlled study of a mixture of volatile organic compounds (VOCs), ozone, and VOC–ozone reaction products (Fiedler et al. 2005, 2008).

In an earlier controlled-exposure study, GWVs reporting illness showed signs and symptoms of stress-induced hyperventilation during an exposure to diesel fuel vapors, including reduced end-tidal CO_2_ (Fiedler et al. 2004). In the present study of healthy young volunteers, we observed no change in end-tidal CO_2_ during DE compared with CA exposures (data not shown), indicating a potentially important difference in stress response between healthy individuals and symptomatic GWVs, which may have reflected higher levels of anxiety among veterans with higher levels of chemical intolerance. The ill GWVs in the diesel vapor study had a mean CII score of 16.3, whereas the healthy GWVs in the earlier study and the subjects in the present study had mean CII scores of 6.7 and 6.6, respectively. In the present study, CII score was not correlated with increased self-reported anxiety during the exposure or reduced end-tidal CO_2_ (data not shown). Despite the relatively low mean CII score, the severity of sickness response symptoms during exposure to DE was greater among subjects with higher CII scores (Figure 4).

*Limitations.* Several factors may limit the ability to generalize our results to naturalistic coexposures to air pollutants and stress. The level of exposure to DE in our study was high relative to ambient air levels, but within the range of concentrations that have been reported in workplace studies (Pronk et al. 2009). Our choices of experimental stressor and temporal relationship to the DE exposure were limited by practical and ethical considerations. We chose a public-speaking task that has been shown to consistently elicit physiological stress responses (Al’Absi et al. 1997). Whereas naturalistic exposures to acute stressors and DE may occur in a multitude of temporal relationships, we chose a concurrent temporal relationship between the stressor and exposure to DE, believing it to be the most straightforward option. Evaluation of conditioned responses, which are of potential relevance to the persistence or chronicity of MUSs, was beyond the scope of this study. The generalizability of our findings to other stressors of different quality, intensity, and temporality is also limited. Various types of experimental and naturalistic stressors have different psychological and physiological correlates (Kusnecov et al. 2001). A strength of our controlled-exposure experimental design is that it allowed us to control for potential confounding exposures to some extent. We did not control for ambient exposures to DE, but the 300-μg/m^3^ exposure concentration was about 100-fold greater than ambient urban levels of DE (U.S. Environmental Protection Agency 2002). Exposure to DE in an exposure chamber, peripheral venous catheter placement, and serial blood draws may have caused background stress that would tend to obscure the effect of the experimental stressor on symptoms. Finally, although our study of 100 subjects was large compared with other similar controlled-exposure studies, the lack of statistically significant three- and four-way interactions in some of our analyses must be interpreted in light of our limited power to detect these effects.

The overall levels of symptom severity and the magnitude of the effect of CII score on sickness response symptoms during DE exposure were relatively small. One factor that might have reduced symptom severity in our study was the constant level of DE exposure, which may have led to adaptation during the exposure session (Prah 1998). In contrast, naturalistic exposures to DE tend to have a high degree of variability over short time frames (seconds to minutes). Concentrated exposures to other irritants over short time periods have been shown to be more potent than lower-level exposures over longer periods (Shusterman et al. 2006). Additionally, the low level and narrow range of CII scores among our subjects may have reduced our ability to see modifying effects of CII score on symptom reporting. The subjects who volunteered to participate in our study had lower mean CII scores (mean ± SD, 6.6 ± 1.9) than did a similar college student community sample (9.5 ± 4.0) that was previously reported (Szarek et al. 1997) or among ill GWVs in our previous study (16.3 ± 5.0). We did not select for CII score, and subjects with higher CII scores were probably less likely to volunteer for exposure to DE.

The pathways underlying the association between increased CII scores and increased reporting of nonspecific symptoms from DE are not known. Because subjects could not be effectively blinded to the odor of DE, we cannot rule out biased reporting by subjects who self-identify as more intolerant of chemicals.

## Conclusions

This study was designed to test a hypothetical model by which acute exposure to DE and stress in susceptible individuals might lead to enhanced acute sickness responses. We observed that DE can cause increased symptomatic illness under controlled conditions, and that CII score may predict heightened sickness symptom responses to DE. These observations demonstrate that DE, and perhaps other noxious air contaminants, can induce acute symptomatic illness responses. According to one hypothetical explanation for MUSs, such unconditioned acute responses to initial higher-level exposures may lead to conditioned responses to odors and other stimuli in everyday life that are reminiscent of the initial exposure. We limited the scope of our study to the acute illness effects coexposure to acute psychological stress and a higher-level exposure to DE. Further study is needed to assess whether, and under what circumstances, such sickness responses might persist.

## Supplemental Material

(20 KB) PDF
