# Supplementary material for: Sickness Response Symptoms among Healthy Volunteers after Controlled Exposures to Diesel Exhaust and Psychological Stress
Source: Environ Health Perspect. 2011 Feb 17;119(7):945–50. doi: 10.1289/ehp.1002631 (PMC3223003; doi:10.1289/ehp.1002631)
Supplement: (20 KB) PDF [file ehp.1002631.s001.pdf]

SUPPLEMENTAL MATERIAL

Sickness Response Symptoms among Healthy Volunteers after Controlled Exposures to Diesel Exhaust and Psychological Stress

Robert J. Laumbach[1], Howard M. Kipen[1], Kathie Kelly-McNeil[1], Junfeng Zhang [2], Lin Zhang [2], Paul J. Lioy[1], Pamela Ohman-Strickland[2], Jing Gong[2], Alexander Kusnecov[3], Nancy Fiedler[1]

University of Medicine and Dentistry of New Jersey – Robert Wood Johnson Medical School, Environmental and Occupational Health Sciences Institute, 170 Frelinghuysen Road, Piscataway, NJ 08854

[1]University of Medicine and Dentistry of New Jersey – Robert Wood Johnson Medical School, Piscataway, NJ, [2]University of Medicine and Dentistry of New Jersey –School of Public Health, Piscataway, NJ, [3]Rutgers, the State University of New Jersey, Piscataway, NJ

Detailed Study Procedures

On the day of each experimental session, subjects reported to the Clinical Center at 8:30 a.m., and a nurse performed a check-in to ascertain that the study inclusion criteria were met. A pregnancy test was given for women. Subjects completed the symptom questionnaire to establish symptoms prior to entering the CEF (Baseline; S-1). Baseline nasal lavage was collected, EKG electrodes were placed, and a trained nurse placed an indwelling, heparinized catheter in the antecubital vein of the non-dominant arm for serial blood collections to assess cortisol. Subjects were escorted to the CEF where two work stations, separated by a Teflon screen, were located. Subjects were seated at a table with a computer monitor and were visible to the experimenter through a one-way window at all times. Each subject wore headphones that allowed the experimenter to communicate with the subject, but prohibited subjects from hearing each other or instructions given to individual subjects. In addition, if one of the subjects was not delivering a speech, music was played through the head phones to mask the sound of the speech given by the other subject. EKG signals were collected continuously throughout the exposure to monitor the subject. To monitor end-tidal CO_2 a sterilized, disposable cannula was inserted approximately ¼ inch into one anterior nare. Subjects were seated in a chair and asked to rest quietly for 30 minutes to allow adaptation to the catheter and the CEF. Subjects completed the symptom questionnaire (CEF Baseline; S-2) and a baseline blood sample was drawn through the catheter (Baseline blood draw). Pre-exposure end-tidal CO_2 was collected for 5 minutes. The exposure period began 30 minutes after the subject was seated in the CEF. During the initial 10 minutes of the exposure, subjects were asked to again rest quietly. End-tidal CO_2 values 10 minutes

after the onset of exposure were collected. When this collection was complete, subjects were told that they could remove the collection canula from their nose. During the next 5 minutes, subjects completed the symptom questionnaire (S-3). Each subject was then asked to perform the vigilance task to maintain a steady state of arousal for the next 5 minutes. For those subjects assigned to the exposure conditions with the psychological stressor, the public speaking task was then administered followed by the symptom questionnaire (S-4). Subjects assigned to exposure conditions without the stressor performed a simple paper and pencil math task for this period of 10 minutes and then completed the symptom questionnaire. Blood samples were collected before and after the stress/math task to assess stress and immune responses to the exposure. After the post stress/math task blood draw, all subjects were allowed to read for 15 minutes after which they completed the symptom questionnaire (S-5). After the exposure period ended, subjects were asked to guess the exposure condition to validate whether they were blind to exposure and to rate their confidence in their guess. Subjects assigned to the speech stressor completed questions regarding their perceived effort in completing the task. Subjects were then escorted to the Clinical Center where the catheter and EKG leads were removed, and they performed the final symptom questionnaire (S-6). Six hours after completion of the exposure, subjects returned to the Clinical Center to complete the symptom questionnaire (S-7), perform induced sputum, and have a blood sample drawn to assess post-exposure responses. At 24 hours post-exposure, subjects again returned to the Clinical Center to perform nasal lavage, give a blood sample, and complete the symptom questionnaire (S-8).

To maintain subject alertness during periods of relaxation, the subject counted the number of times a target colored-square display appeared on a computer screen. At the end of the task the subject reported the number of target color squares counted.
